# Mucormycosis in 2023: an update on pathogenesis and management

**DOI:** 10.3389/fcimb.2023.1254919

**Published:** 2023-09-21

**Authors:** Abdullah Alqarihi, Dimitrios P. Kontoyiannis, Ashraf S. Ibrahim

**Affiliations:** ^1^ Division of Infectious Diseases, The Lundquist Institute for Biomedical Innovation at Harbor-University of California Los Angeles (UCLA) Medical Center, Torrance, CA, United States; ^2^ Department of Infectious Diseases, Infection Control and Employee Health, The University of Texas M.D. Anderson Cancer Center, Houston, Texas, United States; ^3^ Department of Medicine, David Geffen School of Medicine at UCLA, Los Angeles, CA, United States

**Keywords:** mucormycosis, invasive fungal infections, immunosuppression, pathogenicity, DKA, rhizopus, Mucorales, COVID-19-associated mucormycosis

## Abstract

Mucormycosis (MCR) is an emerging and frequently lethal fungal infection caused by the Mucorales family, with *Rhizopus*, *Mucor*, and *Lichtheimia*, accounting for > 90% of all cases. MCR is seen in patients with severe immunosuppression such as those with hematologic malignancy or transplantation, Diabetes Mellitus (DM) and diabetic ketoacidosis (DKA) and immunocompetent patients with severe wounds. The recent SARS COV2 epidemy in India has resulted in a tremendous increase in MCR cases, typically seen in the setting of uncontrolled DM and corticosteroid use. In addition to the diversity of affected hosts, MCR has pleiotropic clinical presentations, with rhino-orbital/rhino-cerebral, sino-pulmonary and necrotizing cutaneous forms being the predominant manifestations. Major insights in MCR pathogenesis have brought into focus the host receptors (GRP78) and signaling pathways (EGFR activation cascade) as well as the adhesins used by Mucorales for invasion. Furthermore, studies have expanded on the importance of iron availability and the complex regulation of iron homeostasis, as well as the pivotal role of mycotoxins as key factors for tissue invasion. The molecular toolbox to study Mucorales pathogenesis remains underdeveloped, but promise is brought by RNAi and CRISPR/Cas9 approaches. Important recent advancements have been made in early, culture-independent molecular diagnosis of MCR. However, development of new potent antifungals against Mucorales remains an unmet need. Therapy of MCR is multidisciplinary and requires a high index of suspicion for initiation of early Mucorales-active antifungals. Reversal of underlying immunosuppression, if feasible, rapid DKA correction and in selected patients, surgical debulking are crucial for improved outcomes.

## Introduction

Fungi are ubiquitously found in many environments, and have important roles in the ecosystem and biodiversity as they are essential in nutrient cycling and recycling of waste (Frąc et al., 2018). It is estimated that there are 1.5 million different types of fungi, from which only 300 are known to cause illness (Centers for Disease Control and Prevention, https://www.cdc.gov/fungal/diseases/index.html). In this review, we focus on Mucorales, a group of commercially and increasingly medically significant molds. Specifically, we provide an overview of the pathogenesis, along with epidemiology (in view of the recent major outbreak of COVID-19 associated mucormycosis in India), pathology and molecular diagnosis, and current therapeutic advances in mucormycosis (MCR).

**Figure 1:**
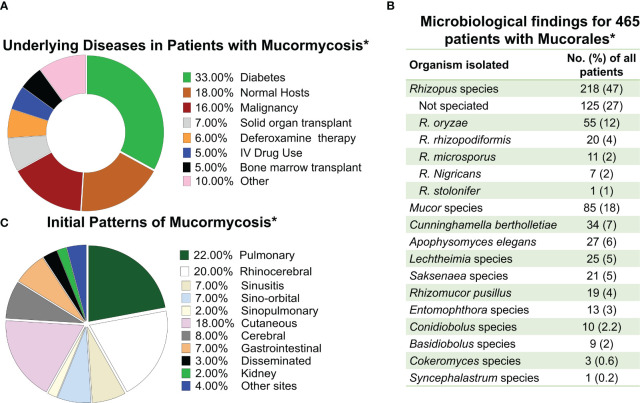
Frequency of mucormycosis manifestation in susceptible hosts and the etiologic agents of the disease. **(A)** Frequency of mucormycosis by underlaying predisposing host condition. **(B)** Etiological agents of mucormycosis. **(C)** Frequency of different types of mucormycosis reported. * Data adapted from Roden M et al. CID 2005 (Roden et al., 2005).

The importance of Mucorales fungi has been established as multifaceted because of their capacity to release a range of commercially used lytic enzymes including amylases, lipases, and proteases, as well as production of essential medical and pharmacological substances such as steroids and terpenoids as well (Morin-Sardin et al., 2017). However, since its first description by Paltauf in 1885 MCR, the invasive disease caused by a variety of Mucorales has come to central stage in modern mycology and infectious diseases. MCR is a severe and frequently lethal infection which can affect a pleiad of different immunosuppressed patients such as those who have received organ transplants and immunocompetent hosts who are trauma victims of natural disasters (Spellberg et al., 2005a; Warkentien et al., 2012; Tribble et al., 2013; Warkentien et al., 2015; Weintrob et al., 2015; Spellberg and Ibrahim, 2018) (**Figure 1A**).

## Nomenclature and frequency of Mucorales as causes of MCR and burden of the disease

MCR is caused by fungi belonging to the order Mucorales. *Rhizopus*, *Mucor*, and *Lichtheimia* (formerly *Absidia*) species are the most common members of the order Mucorales that cause mucormycosis, accounting for >90% of all cases (Uppuluri et al., 2021). *Rhizopus* species, are the dominant cause of MCR in the entire world responsible for >70% of all cases of MCR (Ribes et al., 2000; Roden et al., 2005; Spellberg et al., 2005a). In contrast, *Cunninghamella*, *Apophysomyces*, *Saksenaea*, *Rhizomucor*, *Cokeromyces*, *Actinomucor*, and *Syncephalastrum* species individually are responsible for fewer than 1 to 5% of reported MCR cases (Gomes et al., 2011) (**Figure 1B**). Thus, *Mucor* species, including *M. lusitanicus* (formerly *Mucor circinelloides f. lusitanicus*) (Wagner et al., 2020), and *Lichtheimia* are the secondary cause of infection in the Americas and Europe, respectively. However, *Apophysomyces* are the secondary cause of infection in India (Skiada et al., 2018; Nucci et al., 2019).

Although it is not possible to determine the exact burden of MCR worldwide, there has been an alarming increase in cases in the last three decades. Over the past 15 years cases have more than doubled at the MD Anderson and Fred-Hutchinson Cancer Centers (Kontoyiannis et al., 2000; Marr et al., 2002). According to a scientific study conducted in France, there was a significant increase of 70% between 1997 and 2006. Additionally, there was a substantial rise of 175% in the prevalence of the condition between the years 1988-2006 compared to the period from 2007-2015 (Roden et al., 2005). Also, a medical center in Switzerland reported >10-fold increase in MCR cases among admitted patients after 2003 (Ambrosioni et al., 2010). Cases in Iran more than doubled between 2008-2014 (Dolatabadi et al., 2018). In hematopoietic and allogenic stem cell transplant recipients, MCR is currently the third most prevalent invasive fungal infection, after candidiasis and aspergillosis (Kontoyiannis et al., 2000; Petrikkos et al., 2012). That increase in MCR incidence at many transplant centers has been linked to the introduction and widespread use of voriconazole prophylaxis in these high-risk populations. However, it is not known if this association reflects a true epidemiological link or represents a marker of changing immunosuppression occurring in parallel with the evolution of transplant practices and immunosuppression strategies (Pongas et al., 2009).

Before the onset of the COVID-19 era, India was recognized as hyper-endemic for MCR, a fungal infection. The estimated disease burden in India was approximately 70 times higher than the global average, with predicted cases exceeding 200,000 per year. These cases accounted for approximately 24% of all invasive mold infections (Chakrabarti and Singh, 2014; Chakrabarti et al., 2019; Prakash and Chakrabarti, 2021).

## Morphogenesis

**Figure 2:**
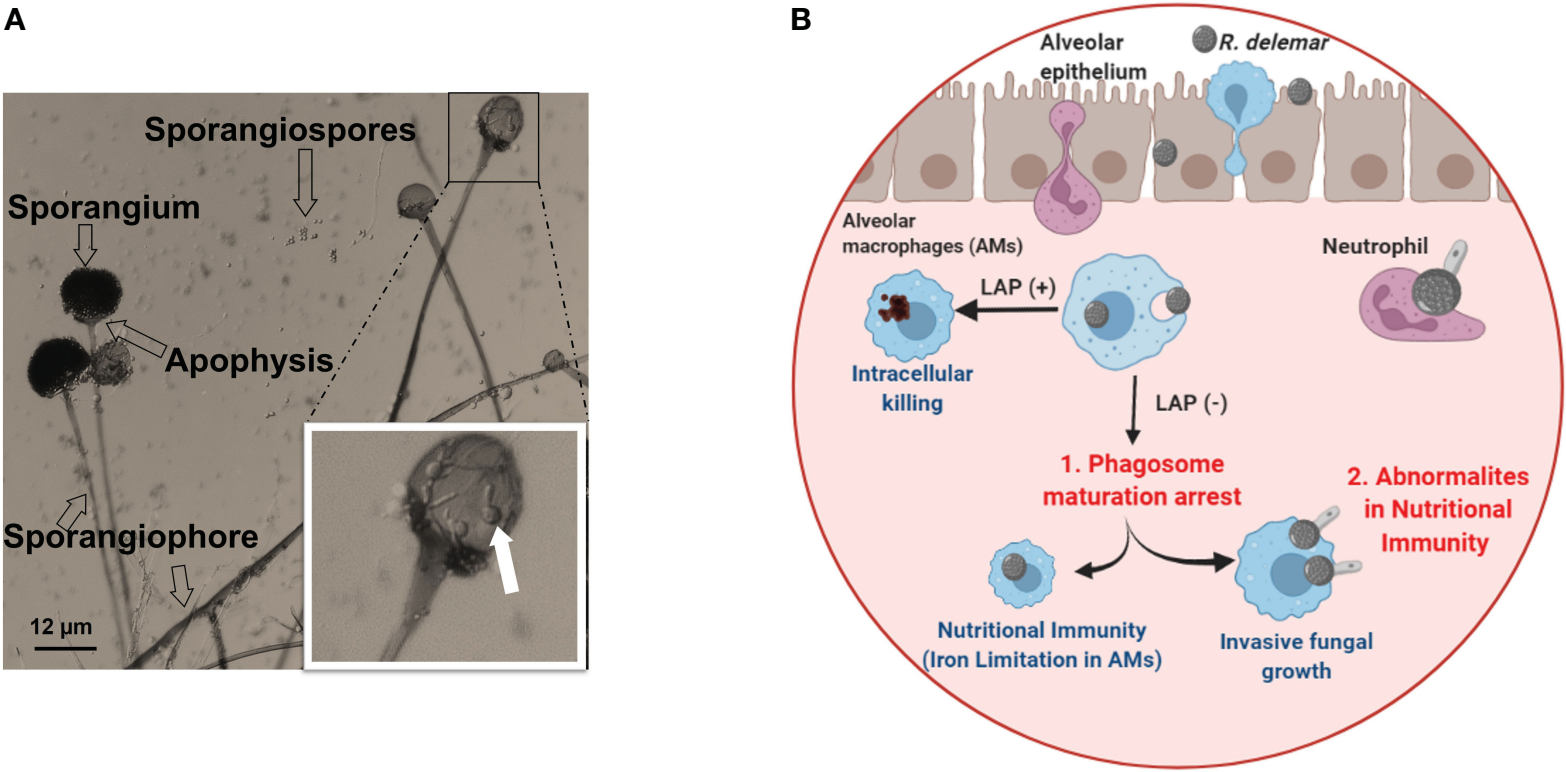
**(A)** Morphology of *Rhizopus delemar*. Sporangia form at the apices of sporangiophores and contain the asexual sporangiospores. Germinated spores seen in the sporangium magnified box can be an overlay of the sporangium on released and germinated spores. **(B)** Under normal circumstance, alveolar macrophages (AMS) are able to phagocytize fungi and killing through LC3-associated phagocytosis (LAP+). While AMS are able to phagocytize Mucorales spores, spore melanin is able to arrest LAP to prevent phagosome maturation. However, spores are unable to grow and germinate due to iron restriction (Frąc et al., 2018). In the presence of abnormal nutritional immunity (*i.e.* excessive iron) spores are able to germinate and kill Ams (Andrianaki et al., 2018). Courtesy of Dr. Georgios Chamilos. “Created with BioRender.com”.

Mucorales fungi can reproduce through both sexual and asexual means. Asexual reproduction involves the formation of spherical structures called sporangiospores, which are located at the tip of the sporangium (as depicted in **Figure 2A**). These sporangiospores can be released and spread, eventually germinating into hyphae. On the other hand, the sexual cycle of most Mucorales fungi begins with the fusion of two opposing types, denoted as (–) and (+), which leads to the formation of zygospores (Lee et al., 2010). These zygospores then germinate and develop into a sporangium at the apex, resulting in the production of sexual meiospores (Lee et al., 2010). Because the sexual life cycle for sporangiospore generation is protracted, anecdotal evidence suggests that asexual sporangiospores may be the primary source of initiation, propagation and spread of infection.

## Host immune responses

### Host barrier cells

Inhalation of spores from the environment causes rhino-orbital/cerebral, sino-pulmonary MCR, the two most common disease manifestations. Cutaneous MCR is the disease’s third most prevalent presentation, and it usually the consequence of inoculation of Mucorales spores to skin/subcutaneous tissues following severe trauma or abrasions on the skin (Sugar, 2005; Ibrahim et al., 2011) (**Figure 1C**). As MCR is characterized by extensive tissue invasion and tissue destruction and is the most angioinvasive of all fungal diseases, dissemination is very common (Ben-Ami et al., 2009).

Mucorales invade nasal and alveolar epithelial cells through binding to host cell glucose-regulated protein 78 kDa (GRP78) and integrin α3β1, respectively (Alqarihi et al., 2020). Watkins et al., 2018 used transcriptome sequencing (RNA-seq) to assess host transcriptional response during early stages of *R. delemar* infection to gain insight into governed Mucorales-host airway epithelial cell early interactions (Watkins et al., 2018). The host epidermal growth factor receptor (EGFR) signaling was activated during infection, and the alveolar epithelial cell EGFR (using A549 cell line) was also phosphorylated when interacting with several Mucorales organisms. Furthermore, the EGFR co-localized with *R. delemar* spores during invasion of alveolar epithelial cells (Alqarihi et al., 2020). EGFR inhibitors cetuximab and gefitinib protected airway epithelial cells from *R. delemar* invasion and injury *in vitro*. These findings identified EGFR activation cascade as a critical pathway in inducing invasion of alveolar epithelial cells by Mucorales and suggested that adjunctive therapy such as gefitinib can be useful in the treatment of pulmonary MCR (Watkins et al., 2018). Finally, Mucorales fungi appear to hematogenously disseminate by engaging the endothelial GRP78 receptor (Liu et al., 2010).

### Innate immunity

The first line of effector immune cells against inhaled Mucorales spores are alveolar macrophages (Aberdein et al., 2013; Ibrahim and Voelz, 2017). Immunocompetent mice have macrophages which are efficient in phagocytizing Mucorales spores and preventing their germination (Diamond and Erickson, 1982; Waldorf et al., 1984a; Waldorf et al., 1984b; Waldorf, 1989). Contrasting to *Aspergillus fumigatus* conidia, macrophages from immunocompetent individuals are unable to kill phagocytized Mucorales spores, despite being able to adhere to Mucorales hyphae and damage them by oxidative and non-oxidative mechanisms (Diamond and Clark, 1982; Waldorf et al., 1984b; Waldorf, 1989; Lee et al., 2015). In contrast to macrophage from immunocompetent hosts, macrophages from diabetic mice cannot prevent spore germination, thereby resulting in established infection and lethality in infected mice (Waldorf et al., 1984b). Unraveling the enigma of alveolar macrophages’ enduring presence and their resilience against destruction could be essential in formulating innovative approaches for combating infections. Andrianaki et al., 2018 discovered that alveolar macrophages-phagocytized Mucorales spores retain melanin on their surface and therefore are able to halt phagosome maturation through inhibition of LC3-associated phagocytosis (Andrianaki et al., 2018). Furthermore, research employing transcriptome, iron supplementation, and genetic modification of iron acquisition genes revealed that iron restriction inside macrophages modulates immunity against *Rhizopus* and suppresses fungus germination (**Figure 2B**) (Andrianaki et al., 2018). Future studies are destined to shed more light into the important role of nutritional immunity to control Mucorales.

Mucorales are resistant to innate immune cells with hyperglycemia impairing chemotaxis and killing activities of polymorphonuclear cells, including neutrophils in the DKA settings (Chinn and Diamond, 1982). As acidosis impairs transferrin’s ability to efficiently chelate iron (Artis et al., 1982; Ibrahim et al., 2008b; Kontoyiannis et al., 2012; Gebremariam et al., 2016), the released iron causes further functional impairment in phagocytes (Cantinieaux et al., 1999; Guo et al., 2002). Immune cells from mice fed excessive amounts of iron secrete a reduced amount of interferon-gamma (IFN-γ) (Omara and Blakley, 1994), a signature cytokine that orchestrates Mucorales fungal death by effector immune cells (Gil-Lamaignere et al., 2005). IFN-γ and granulocyte-macrophage-colony-stimulating factor (GM-CSF) have specifically, either alone or combined, shown to improve neutrophils’ ability to damage and kill Mucorales hyphae *ex vivo* by increasing oxidative burst and TNF-α release (Gil-Lamaignere et al., 2005).

### Adaptive immunity

The role of adaptive immunity in MCR patients has not been extensively investigated. Similar to the interaction with *Aspergillus*, the exposure to β-glucan during the germination process of Mucorales fungi triggers dectin-1 signaling in human dendritic cells. This signaling pathway leads to the strong activation of IL-23 and Th-17 responses, similar to the immune responses observed in the presence of *Aspergillus* (Chamilos et al., 2010). Hyphae can be destroyed in patients who elicit Mucorales-specific T-cells (Potenza et al., 2011; Schmidt et al., 2013). Consistent with these results, T-cells that have been pulsed with *Rhizopus* extract and stimulated with IL-2/IL-7 produce Mucorales-specific T cells with CD4+ cells (Castillo et al., 2018). Instead of non-specific signaling, these cells can produce IFN-γ, IL-5, IL-10, IL-13, and TNF-α and detect fungus antigens processed by HLA-II molecules (Castillo et al., 2018). In addition, emerging evidence points out of the potential role of the benefit of adjunct immune checkpoint inhibitors (ICIs) to treat MCR. In a proof-of-concept study, Wurster et al. studied the effects of PD-1 and PD-L1 inhibitors outcomes and immunopathology of invasive pulmonary MCR in cyclophosphamide- and cortisone acetate-immunosuppressed mice. *R. arrhizus*-infected mice receiving either of the both PD-1 but even more so by PD-L1-inhibitor (without concomitant antifungals) had significantly improved survival, less morbidity, and lower fungal burden compared to isotype-treated infected mice (Wurster et al., 2022). As inhibition of the PD-1/PD-L1 pathway is not without the potential for immune-related adverse events, future careful dose-effect studies are needed to define the “sweet spot” between ICI-induced augmentation of Mucorales immunity and potential immunotoxicities.

## Pathogenicity factors

### Hyphal formation

In response to environmental stimuli, Mucorales can rapidly switch their morphogenetic programs between spores, and mycelia (Orlowski, 1991). Yeast-like form development in *Mucor* spp. are promoted by the presence of fermentable hexose and Anaerobiosis, whereas oxygen and nutrient constraint promote hyphal growth (Wolff et al., 2002). Gene targets that modulate morphogenesis were identified with gene deletion homologous recombination techniques with autotrophic markers, and this information has led to intriguing therapeutic candidates. specifically, the calcineurin pathway, for example, was found to govern yeast to mycelium transition and influenced pathogenicity in *M. lusitanicus* (Lee et al., 2015). Specifically, the chemical inhibition of calcineurin or the disruption of the regulatory subunit gene of calcineurin (CnbR) traps the yeast-form, specifically, making it substantially less virulent in mice.

In addition to calcineurin, cyclic AMP (cAMP) and its target protein kinase A (PKA) are thought to have a role in morphogenesis control. A cross-talk between these two regulatory mechanisms is implied, calcineurin inhibits PKA (Lee et al., 2013). Further gene deletions uncovered other proteins involved in the control of *M. circinelloides* dimorphism, such as heterotrimeric G proteins and ADP-ribosylation factors (Arfs) (Patiño-Medina et al., 2018). Finally, a role in pathogenicity is thought to be played by the size of Mucor spores. Specifically, in *Galleria mellonella larvae* MCR model large multinucleate spores germinate faster and are more virulent than small mononucleate spores (Li et al., 2011). Small spores are phagocytized more avidly, whereas larger spores can geminate inside macrophages and overpower them.

### Effect of iron homeostasis

Like all pathogens, iron is crucial for Mucorales survival in the host. Mammalian cells store iron bound to iron-carrying proteins such as ferritin, lactoferrin and transferrin (Howard, 1999). DKA or other types of acidosis, elevated blood glucose and low blood pH, in patients with hypoglycemia, disturb the avidity of these host proteins to bind iron, resulting in an increase in serum free iron concentration (Artis et al., 1982; Ibrahim, 2014; Gebremariam et al., 2016). Increased availability of serum free iron levels in the host can enhance the ability of Mucorales to produce a rapidly invasive infection (Boelaert, 1994; Boelaert et al., 1994; Ibrahim et al., 2007). A high-affinity iron absorption system and the production of siderophores are used by Mucorales to acquire exogenous iron by these two mechanisms (Carroll et al., 2017; Navarro-Mendoza et al., 2018; Lax et al., 2020). A family of iron reductases (Fre), a ferroxidase (Fet3), and a high affinity iron permease (Ftr1) make up the high-affinity iron acquisition system (Navarro-Mendoza et al., 2018). In *R. delemar* (Fu et al., 2004; Ibrahim, 2010; Liu et al., 2015), *M. circinelloides* (Navarro-Mendoza et al., 2018), and *L. corymbifera*, low iron availability stimulates the development of the high-affinity iron absorption system (Schwartze et al., 2014). The functions of genes related with the iron uptake in Mucorales was explored using the RNAi gene silencing, particularly in *R. delemar* (formerly identified as *R. oryzae*), which is less amenable to mutagenesis than *M. circinelloides* (Ibrahim, 2010). In mice with DKA, reduction of the copy number of the *FTR1* gene or inhibition of expression by RNAi impairs *R. delemar*’s ability to accumulate iron *in vitro* and lower its pathogenicity (Ibrahim, 2010).

Historically, patients on hemodialysis taking deferoxamine to treat iron overload toxicity had a very high risk for disseminated and frequently lethal MCR (Boelaert et al., 1987; Boelaert et al., 1989; Boelaert et al., 1991; Boelaert, 1994). For its growth, *R. delemar* uses iron from ferrioxamine as a siderophore (the iron rich form of deferoxamine). According to biochemical and genetic investigations (by RNAi-mediated gene silencing) *R. delemar* has two surface receptors (Fob1 and Fob2) that bind ferrioxamine and enhance iron intake via a reductase/Ftr-1 mediated pathway (Liu et al., 2015). In addition, three ferroxidase encoding-genes have been identified in *M. circinelloides*: fet3a, fet3b, and fet3c (Navarro-Mendoza et al., 2018). All three *M. circinelloides* genes are overexpressed in the lungs of infected mice, and they are regulated by iron availability in the culture media. In addition, there is a relationship of the expression of the different ferroxidase with the fungal dimorphic state, a key virulence factor for Mucorales invasion. Specifically, during aerobic growth, fet3a is expressed specifically in yeast-like growth, while fet3b and fet3c are expressed in hyphae (Navarro-Mendoza et al., 2018). As proof of concept, gene deletion studies revealed that fet3c plays a major role in *M. circinelloides* virulence *in vivo* (Navarro-Mendoza et al., 2018).

In addition to using the bacterial deferoxamine as a xenosiderophore, *Rhizopus* species are known to synthesize and secrete their own Rhizoferrin siderophore (Carroll et al., 2017). This siderophore supplies *Rhizopus* with iron through a receptor-mediated, and energy dependent process (Thieken and Winkelmann, 1992; de Locht et al., 1994). A *R. delemar* rhizoferrin synthetase (SfnaD), a gene that has homology to the bacterial NRPS-independent siderophore (NIS) protein has been identified in a study (Carroll et al., 2017). The SfnaD contains the C-terminus conserved ferric iron reductase FhuF-like transporter domain. In addition, growing the fungus in iron-rich conditions inhibited the expression of SfnaD in *R. delemar*, while heterologous expression of this gene allowed *E. coli* to synthesize the siderophore from citrate and diaminobutane, thereby confirming its identity as a NRPS-independent siderophore protein. It is important to mention that rhizoferrin was shown to be inefficient in chelating iron from serum (Boelaert et al., 1993; Boelaert et al., 1994). Consequently, the role of rhizoferrin in *Rhizopus* virulence might be limited in hosts that do not have excess free-iron, while this siderophore could be operative in hosts with elevated free-iron such as DKA patients. In fact, iron regulation is quite complex in Mucorales. It depends on the specific Mucorales genus and species and the particular context (low or high iron availability).

Finally, the genome project of Mucorales fungi revealed the presence of gene orthologs to heme oxygenase which were shown to be important in acquiring iron from haemin in several fungi (Worsham and Goldman, 1988; Santos et al., 2003). It is possible that these heme oxygenase genes are involved in acquiring iron from host hemoglobin and might explain the angioinvasive nature of the disease (Ibrahim et al., 2008b; García-Carnero and Mora-Montes, 2022).

### CotH significant role in invasion

Mucorales interact actively with epithelial cells and the endothelium lining blood vessels to promote initiation of infection and angioinvasion, respectively. Among the fungal kingdom found only in Mucorales are spore coating proteins (CotH) family members, which are kinase-like proteins (Chibucos et al., 2016; Gebremariam et al., 2016; Nguyen et al., 2016). CotH proteins were first described in *Bacillus subtilis* in which it is involved in endospore formation. The function of CotH proteins in Mucorales is to mediate invasion of host cells, including epithelial and endothelial cells (Gebremariam et al., 2014; Gebremariam et al., 2016; Alqarihi et al., 2020). CotH proteins were also found to be required for normal spore formation and virulence in *M. lusitanicus* (Szebenyi et al., 2023). The number of CotH genes is correlated to the pathogenic potential of agents of MCR, with the genera most implicated in invasive infections (*Rhizopus, Mucor*, and *Lichtheimia*) (Warkentien et al., 2012; Warkentien et al., 2015; Weintrob et al., 2015) having multiple copies of the CotH (Chibucos et al., 2016). CotH3 and CotH7 (called collectively “invasins”) promote invasion of nasal and alveolar epithelial cells through binding to host cell GRP78 and integrin α3β1, respectively (Alqarihi et al., 2020). The mechanism by which CotH7 binds to integrin α3β1 on alveolar epithelial cells initiates invasion of the host is believed to be related to activation of EGFR which initiates a cascade of events involved in endocytosis (Watkins et al., 2018).

In addition to epithelia, CotH3 is also involved in promotion of angioinvasion through attaching to GRP78 on endothelial cells (Gebremariam et al., 2014; Gebremariam et al., 2016). GRP78 is a heat-shock protein belonging to the HSP70 family that is expressed on the mammalian cell membrane in response to various stressors (Lee, 2007; Alqarihi et al., 2020). Furthermore, serum iron increases in availability, when key host conditions were encountered in DKA such as hyperglycemia and the presence of ketone bodies (e.g., β-hydroxy butyrate), have all been shown to create a “perfect storm” of increased and rapid invasion and robustly increase the expression of both GRP78 and CotH3 in the target organs of mice with DKA (Liu et al., 2010; Gebremariam et al., 2016; Alqarihi et al., 2020). Furthermore, the density of GRP78 receptors is richer in sites where MCR is most common. Thus, GRP78 high expression on invaded endothelial cells and macrophages in necrotic tissues were revealed through the immunohistochemistry of the ethmoidal sinus tissue of a patient with rhino-cerebral MCR (Shumilov et al., 2018). Interestingly, *R. delemar*’s capacity to invade and damage endothelial cells *in vitro* and reduce disease severity in mice is diminished by the activity of CotH3 proteins blocking their activity, either by using anti-CotH monoclonal antibodies or by genetically attenuating CotH3 expression (Gebremariam et al., 2016). As a result, the distinctive susceptibility of patients with DKA to MCR is explained by the unique and concomitant amplification of interaction between CotH3 and GRP78 under hyperglycemic/ketoacedotic settings.

Of critical importance is the ability of an anti-CotH3 monoclonal antibody to block host cell invasion and ameliorate the disease in mice when given alone after infection. Further, this antibody demonstrates synergy when given with antifungal drugs to treat severe murine MCR (Gebremariam et al., 2019). A humanized version of the antibody was also shown to equally protect against the disease in mice and is currently in manufacturing (Gu et al., 2021).

### Mycotoxins

**Figure 3:**
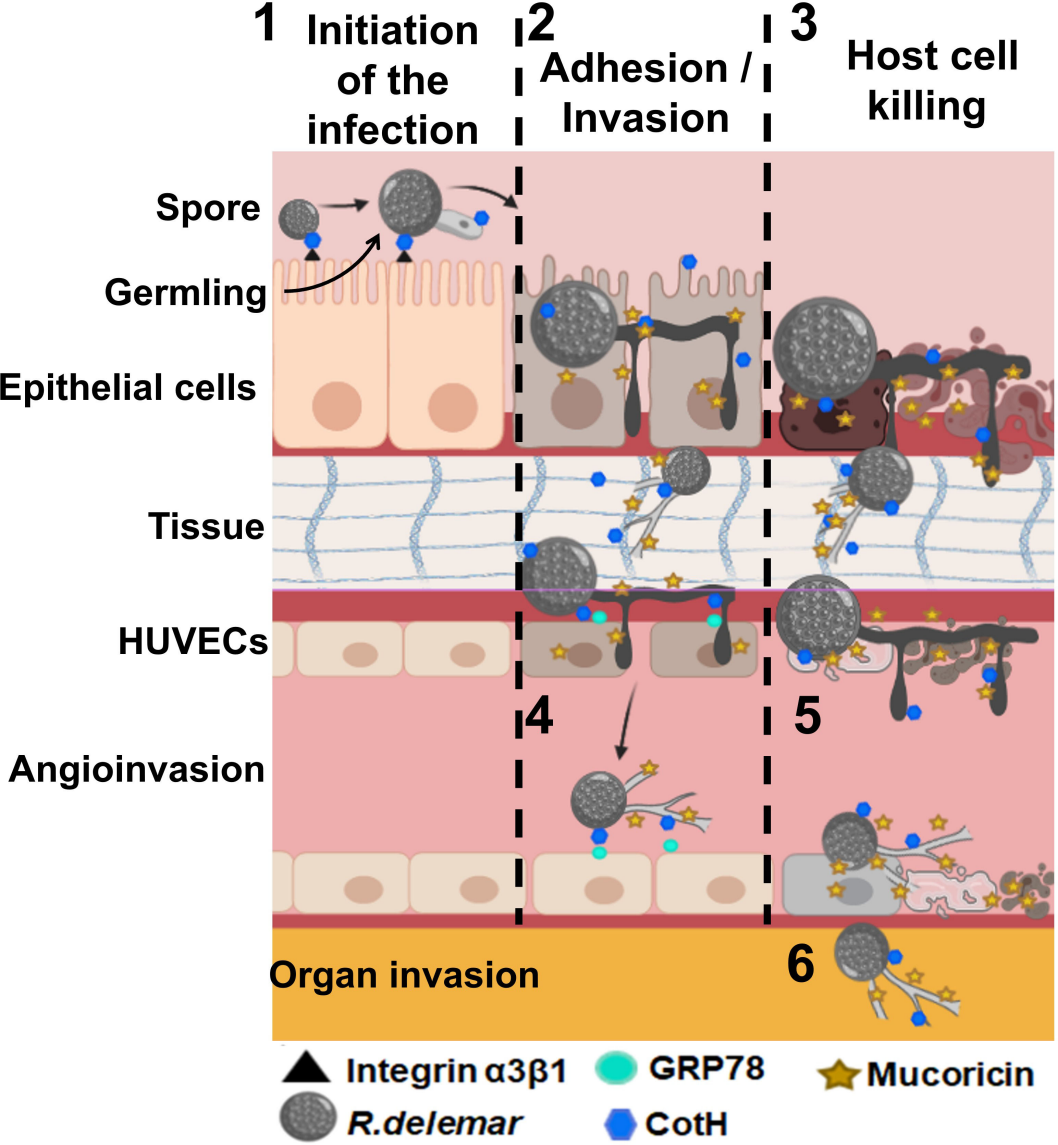
Postulated events that lead to adhesion and invasion and host cell death. 1) Inhaled spores bind to epithelial via CotH/integrin a3β1 followed by germination. Germlings produce mucoricin. 2) Considerable disruption of the epithelium due to invasion of the spores within hours of infection. 3) Mucoricin causes host cell death within 48 h of infection. 4) Angioinvasion of hyphae and sporulated cells occur *via* CotH and endothelial cell GRP78 interactions, 5) endothelial cell injury occurs after infection with *R. delemar* spores resulting in tissue necrosis. 6) Hematogenous dissemination results in organ seeding. Tissue edema and organ failure are the results of excessive vascular leak. “Created with BioRender.com”.

The first evidence of the presence of toxins in Mucorales came from the observation that even killed Mucorales spores were able to cause significant damage to host cells (Ibrahim et al., 2005b). This was followed by a study connecting food poisoning outbreak with Chobani yogurt to *M. circinelloides* (Lee et al., 2014). Recently, Soliman et al. revealed that Mucorales harbor a ricin-like toxin protein of 17 kDa that is expressed during hyphal formation. The toxin has structural similarity to ricin B chain and functionally resembled ricin A chain in blocking host protein synthesis through ribosomal inactivation. Thus, this toxin was named “mucoricin”, and was shown to be critical for MCR pathogenesis in mice. In addition, mucoricin was found to be expressed in lung tissues from a patient with pulmonary MCR (Soliman et al., 2021). Importantly, polyclonal antibodies targeting mucoricin were shown to protect mice from MCR (Soliman et al., 2021) suggesting that further development of immunotherapies against the toxin are likely to aid in managing patients with MCR. Other unidentified toxins are likely to exist, since attenuation of mucoricin expression reduced, but not abrogated, the ability of *Rhizopus* to damage host cells (Soliman et al., 2021). **Figure 3** summarizes pathogenicity events involved in host cell invasion and tissue damage during MCR.

## Genomic structure and genetic manipulation to understand pathogenicity

The availability of restricted tools for genetic manipulation has been a major hindrance to the in-depth research of Mucorales fungus genes and signaling cascades. Early in the Mucormycotina lineage, a whole-genome duplication occurred, and the duplication of genes may have produced novel proteins, so expanding the sensory and signaling pathways (Ma et al., 2009; Schwartze et al., 2014; Garcia et al., 2018). When sexually reproducing, Mucorales are known to be haploid and display zygotic meiosis (Morin-Sardin et al., 2017). *Mucor*, and *Rhizopus*, and *Lichtheimia* species are the only Mucorales known so far to be amenable to genetic manipulation. Due to the paucity of dominant selection markers, genetic experimentation even with these two fungi is difficult, the limited transportation efficiency, and the rarity of chromosomal integration. RNA interference (RNAi) is the most often used approach as a result, rather than disrupting genes (Ibrahim et al., 2010; Calo et al., 2014; Liu et al., 2015; Trieu et al., 2017). Genes involved in virulence and resistance to treatment have been identified by researchers through gene silencing. However, a major drawback of the RNAi includes the possibility of having false positive outcomes due to off-target effects (Schmitt, 2012). In *Mucor*, gene deletion mutants are achieved by targeted integration using either dominant selection or auxotrophic markers (Appel et al., 2004; Larsen et al., 2004; Nicolas-Molina et al., 2008).

Vital information on the involvement of the calcineurin pathway in MCR pathogenesis has been yielded through gene disruption by homologous recombination effectively applied in *M. circineloides* (Lee et al., 2013; Lee et al., 2015). The CRISPR/Cas9 system for gene editing of genomic DNA is the most recent advancement in molecular tool development. In order to disrupt a toxin-encoding gene in *R. delemar* utilizing a single plasmid with pyrF as a marker and the biolistic delivery system this approach was first used in Mucorales (Baldin et al., 2017). Southern blot analysis, abrogation of toxin expression, and a dramatic reduction in *R. delemar’s* ability to kill host cells all verified gene disruption (Baldin et al., 2017). Consistent with CRISPR-Cas9- induced gene mutation by non-homologous end joining (NHEJ) the CRISPR/Cas9 has been utilized to create *R. delemar* pyrF mutants with a single nucleotide deletion at the fourth nucleotide before the protospacer adjacent motif (PAM) sequence (Bruni et al., 2019). The CRISPR-Cas9 targeted integration method was also recently adapted to reliably and stably transform protoplasts of *R. microsporus* (Lax et al., 2021; Lax et al., 2022). In *M. lusitanicus*, the CRISPR-Cas9 system has been successfully employed in a plasmid-free way to disrupt two genes: carB, which encodes phytoene dehydrogenase, and hmgR2, which encodes 3-hydroxy-3-methylglutaryl-CoA reductase (Nagy et al., 2017). More recently, uracil auxotrohic strains of *Lichtheimia corymbifera* were obtained by targeted mutagenesis using CRISPR-Cas9 (Ibragimova et al. 2020) These tools show promise in deciphering the role of various regulatory genes during host-pathogen interactions.

## Diagnosis

Early diagnosis of MCR remains a major unmet need as clinical & radiologic predictors lack sensitivity and specificity (Farmakiotis and Kontoyiannis, 2016). Cultures are often negative in tissues (up to 55-75% (Tarrand et al., 2005) as is often immunohistochemistry (IHC)/*in situ* hybridization (Lockhart et al., 2021). There are limited promising new antifungals with Mucorales activity (Lamoth et al., 2022). Thinking of the disease and having a low threshold of initiating therapy that has activity against Mucorales is very important. In patients with hematological malignancies, delaying amphotericin B-based therapy beyond 5 days after onset of symptoms doubles 12-week mortality (Chamilos et al., 2008). Early detection of MCR is critical for timely treatment implementation as a result (Spellberg et al., 2012c). The traditional diagnosis of MCR relies on culturing the organism from normally sterile body locations and/or tissue histology because there is currently no serology test for diagnosis of MCR (Spellberg et al., 2005a; Ibrahim et al., 2012; Cornely et al., 2019). Fungal elements are usually stained with Gomori methenamine-silver, hematoxylin and eosin (H&E), periodic acid-Schiff (PAS), or calcofluor white stain and Mucorales can be isolated on Sabouraud-dextrose agar incubated at 25–37 °C (Lass-Flörl, 2009). However, these procedures are insufficiently sensitive and frequently result in a misdiagnosis due to: 1) possible contamination of the plates, given the widespread nature of Mucorales fungi, cultures can result in false positives; and 2) lack of growth as a result of laboratory mishandling of specimens (*e.g.* homogenization can damage hyphal components and kill the fungus) resulting in false negatives (Spellberg and Ibrahim, 2015). The paradox of the poor recovery of Zygomycetes hyphae from tissue specimens remains unclear, and it may result from failure of current culture methods to mimic physiologic conditions found in hyphae-laden infected. Experimental evidence suggests that simulating Mucorales growth under necrotic or semi-anaerobic tissue conditions enhances culture yield (Kontoyiannis et al., 2007). Even the “gold standard” that of histopathology detection of the characteristic nature of the board ribbon-like aseptate Mucorales hyphae is far from perfect. Minimal influence on the outcome of therapy can be achieved with definitive histologic identification based on morphology which can lead to error and often occur at a late stage of the infection (Dadwal and Kontoyiannis, 2018). Radiological clues, in the appropriate clinical context, make the early suspicion of MCR possible in patient with hematologic cancer. The illness may be distinguished from invasive pulmonary aspergillosis through the radiological clues in chest CT along with the presence of a reversed halo sign in patients with a hematologic malignancy or neutropenia that have a strong predictive value for MCR (Chamilos et al., 2005; Spellberg et al., 2005a; Georgiadou et al., 2011; Legouge et al., 2014; Jung et al., 2015). Thus, the development of polymerase chain reaction (PCR)-based technologies and Matrix Assisted Laser Desorption/Ionization-Time of Flight Mass Spectrometry (MALDI-TOF MS) which are recent advancements in molecular diagnostics, have generated significant excitement the potential to speed up both the diagnosis and treatment of MCR (see below).

### Molecular diagnostics

A potential useful tool for diagnosing MCR are real-time PCR-based approaches, particularly in lung infections caused by *Lichtheimia, Mucor, Rhizopus*, and *Rhizomucor* spp (McCarthy and Walsh, 2016; McCarthy et al., 2017). PCR based diagnosis is particularly promising, in view of the early and rapid dissemination of MCR (in contrast to Aspergillus) The PCR amplification of CotH3, a Mucorales-specific protein, has demonstrated to be specific and sensitive for MCR diagnosis (Baldin et al., 2018). CotH3 was not effectively amplified from urine, plasma, and bronchoalveolar lavage collected from mice infected with *Aspergillus* fumigatus but was effectively amplified in Mucorales fungi-infected mice (Baldin et al., 2018). To identify DNA from the most common MCR agents a multiplex real-time PCR (MRT-PCR) technology has also been created (Nagao et al., 2005; Walsh, 2012). Several studies showed significant inter-laboratory standardization and several supportive retrospective studies and preclinical data correlating MCR burden with qPCR kinetics (Millon et al., 2013; Caillot et al., 2016; Millon et al., 2016; Springer et al., 2016). The nucleotide sequence of the ITS1 ribosomal DNA region from strains belonging to *R. oryzae* and *R. microsporus*, as well as the sequence of the ITS2 region for *Mucor* spp. belonging to *M. circinelloides, M. racemosus, M. plumbeus, and M. velutinosus* (Bernal-Martinez et al., 2013), were used to design primers and molecular probes (Springer et al., 2016). In tissue and serum samples from patients with rhinoorbital/cerebral MCR, a semi-nested PCR-based technique amplifying the 18S region of rDNA unique to Mucorales was shown to be more reliable than ITS2 PCR in identifying infection (Zaman et al., 2017). For circulating Mucorales identification in patients with proven MCR, other quantitative PCR approaches have investigated a mix of hydrolysis probes targeting *Mucor, Rhizopus*, *Lichtheimia*, and *Rhizomucor* (Millon et al., 2013). In 9 out of 10 blood samples from individuals diagnosed with the condition Mucorales DNA was found, which indicates that quantitative PCR might be a valuable screening technique in high-risk patients (Caillot et al., 2016). These data have been further validated in a recent multicenter Study (MODIMUCOR study) (Millon et al., 2022). Mucorales have been identified using other promising technologies such as MALDI-TOF MS (Yaman et al., 2012). However, MALDI-TOF MS has yet to be proven as a reliable method for detecting MCR in clinical samples. As a diagnostic tool the requirement for fungus culture prior to identification, as well as the lack of substantial libraries for uncommon Mucorales has been restricted (Cornely et al., 2019). Other emerging technologies are multiplex pan mold PCR (Alanio and Bretagne, 2017), and a sequence-based identification/shot gun metagenomics (Hoang et al., 2022).

## Treatment of MCR

Reversal of underlying poor prognostic factors such as neutropenia, hyperglycemia, low threshold of suspicion and early initiation of effective antifungal therapy and in selected cases, surgical debridement of affected tissues, and antifungal therapy, are the cornerstones in the management of MCR (Kontoyiannis and Lewis, 2011; Chitasombat and Kontoyiannis, 2016).

### Surgical intervention

To resect all necrotic regions, surgical debridement should be carried out as soon as feasible and should be thorough. In cases of rhino-orbital/cerebral infection surgeries sometimes deformity can result (Vironneau et al., 2014). Better outcomes have been associated with extensive and repeat surgical debridement of the rhino-orbital/cerebral MCR which have been shown by many uncontrolled investigations (Gil-Lamaignere et al., 2005; Chamilos et al., 2010; Potenza et al., 2011). After radical surgery, 90% of patients achieved local infection control, compared to 41.6% in patients who underwent less extensive surgery (Vironneau et al., 2014). However, in modern times, such disfiguring surgeries are much less common in the era of early rhinoscopic evaluation of the sinuses (Davoudi et al., 2015). In pulmonary MCR, although patient selection also plays a role (patients with terminal underlying disease are typically excluded from surgery), studies have shown that patients treated with a combined medical-surgical approach had a better outcome than patients who did not undergo surgery (Lee et al., 1999).

### Treatment with antifungal drugs

*In vitro* data indicate that a limited number of FDA-approved antifungals (amphotericin B-based formulations, and the triazoles posaconazole. isavuconazole and possibly itraconazole) have activity against Mucorales and that there are species-specific differences in susceptibility to azoles (e.g., high posaconazole minimum inhibitory concentrations [MICs] in some *Mucor* species, high MIC to isavuconazole in *Rhizomucor*, multidrug resistance [MDR] in *Cunninghamella* and in some *Rhizopus* spp.) (Almyroudis et al., 2007; Lamoth and Kontoyiannis, 2019; Borman et al., 2021). However, despite the useful information of *in vitro* studies the *in vitro/in vivo* correlation in the management of complicated opportunistic mold infections in humans such is MCR remains problematic (Lamoth et al., 2020).

**Figure 4:**
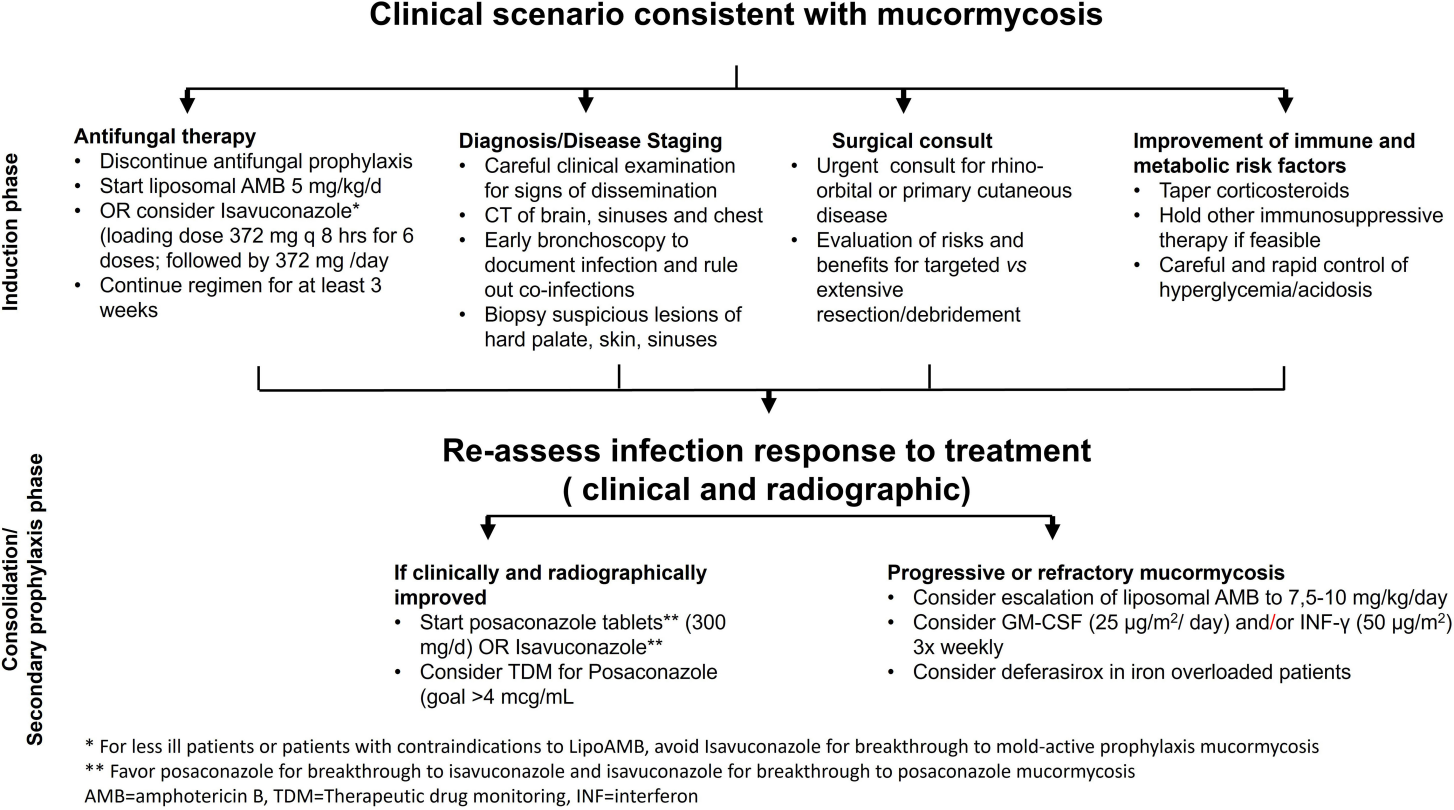
An Algorithm for Mucormycosis Treatment.

Most of the treatment experience is derived from amphotericin B-based therapy. The therapy of choice has become Lipid amphotericin B formulations (*e.g.* liposomal amphotericin B [L-AMB], and amphotericin B lipid complex [ABLC]) since they can be given in higher doses than amphotericin B-deoxycholate and have improved nephrotoxicity index (Fera et al., 2009; Vehreschild et al., 2013; Cornely et al., 2019). As a “step-down” treatment following the primary therapy with L-AMB, the broad-spectrum oral triazoles of posaconazole and isavuconazole can be used (Kontoyiannis and Lewis, 2011; Allen et al., 2015; Marty et al., 2016). There are no randomized clinical trials evaluating the effectiveness of antifungal medications since MCR is an uncommon condition, affecting many host groups, presenting with different clinical syndromes, and caused by a variety of Mucorales (**Figure 1**). However, a multicenter open-label single-arm research (VITAL study) including 37 patients with MCR found that isavuconazole monotherapy is as effective as L-AMB or L-AMB plus posaconazole (Marty et al., 2016). In fact, clinically relevant dosages of isavuconazole, which is licensed by the FDA and the European Medicines Agency for the treatment of MCR patients were equated to tissue clearance and survival in mice (Gebremariam et al., 2020). **Figure 4** introduces an algorithm for MCR treatment.

The poor outcomes of MCR with currently available monotherapy, particularly in patients with hematologic malignancies, has stimulated interest in studying various combinations of antifungal agents (Spellberg et al., 2012b). In contrast to L-AMB + posaconazole combination (Ibrahim et al., 2009), comparative effectiveness of isavuconazole + L-AMB in treating diabetic mice, showed synergy in treating mice infected with either *R. delemar* or *M. circinelloides* (Gebremariam et al., 2021). Given the lack of easy extrapolation from experimental models to the complexity of human MCR, the synergy between L-AMB and isavuconazole therapy is yet to be determined in clinical trials.

Despite harboring the echinocandin-target enzyme glucan synthase (FKS) needed for 1,3-β-glucan production (Ma et al. 2009), Mucorales species show innate *in vitro* resistance to echinocandins because glucans are not a major component of the Mucorales cell wall (Ibrahim et al., 2005a). However, a synergistic relationship between echinocandins and lipid formulation amphotericin B was observed in DKA mice infected with *Rhizopus* showing enhanced survival when compared to monotherapy (Spellberg et al., 2005b; Ibrahim et al., 2008a). These experimental studies are consistent with data obtained from a retrospective study employing limited 41 diabetic patients with rhino-orbital MCR. Specifically, patients treated with a combination of caspofungin and amphotericin B-based drugs had better survival than those treated with amphotericin B-based drugs alone (Reed et al., 2008). It is thought that immune detention and phagocytosis of invading hyphae is allowed when immunological epitopes on Mucorales cell wall, which are unmasked by echinocandins inhibition of *β-*glucan synthesis (Reed et al., 2008). However, in MCR patients with hematologic malignancies and hematopoietic cell transplant recipients, a retrospective cohort study using propensity score analysis found that the combination L-AMB with posaconazole (suspension) or with echinocandins, or posaconazole with echinocandins resulted in no differences in 6-week mortality between monotherapy and combination treatment (Kyvernitakis et al., 2016). It was revealed that L-AMB + micafungin treatment provided little enhancement in survival of neutropenic mice versus L-AMB monotherapy which are in line with data obtained in neutropenic patients (Ibrahim et al., 2008a). Overall, a combination of lipid formulation amphotericin B and echinocandins have been shown by the human retrospective and murine experimental results to help patients with DKA than those with hematologic malignancies or hematopoietic cell transplant recipients. It would be of interest to revisit the merits of combination therapy, with other conventional or investigational antifungals.

### Antifungal drugs in development

Some investigational drugs are currently in development with demonstrated *in vitro* activity against Mucorales and in experimental models of MCR. In a delayed treatment model of immunosuppressed mice infected with *R. arrhizus* var. *arrhizus*, the 1-tetrazole fungal-specific 14 a-lanosterol demethylase (CYP51) inhibitor VT-1161 demonstrated equivalent effectiveness to high dosage L-AMB (Gebremariam et al., 2015). When administered as prophylaxis, both VT-1161 and posaconazole enhanced lifespan and reduced tissue fungal load in immunosuppressed mice infected with *R. arrhizus* var. *arrhizus*. Furthermore, VT-1161 was superior to posaconazole in terms of extending mice survival time when used as a continuous treatment (Gebremariam et al., 2015). Future clinical studies are needed to evaluate the therapeutic impact of tetrazoles in human MCR.

Manogepix (formerly APX001A and E1210) is a first-in-class antifungal drug with good activity against several fungal pathogens including Mucorales fungi (Shaw and Ibrahim, 2020; Lamoth et al., 2022). The fungal Gwt1 enzyme which catalyzed inositol acylation is inhibited by manogepix, an early step in the glycosylphosphatidylinositol (GPI)-anchor biosynthesis pathway (Umemura et al., 2003). Treatment with fosmanogepix (the prodrug of manogepix) substantially enhanced and prolonged median survival time of mice infected with either *R. arrihzus* var. *arrhizus* or *R. arrihzus* var. *R. delemar*, when compared to placebo. Furthermore, a 1-2 log decrease in both lung and kidney fungal loads resulted from fosmanogepix treatment (Gebremariam et al., 2020). Further, a combination of fosmanogepix and L-AMB was found to be superior to monotherapy in treating immunosuppressed mice infected with *R. arrihzus* var. *R. delemar* with enhanced survival, tissue fungal clearance and histology improvement of infected lungs (Gebremariam et al., 2022). Beyond mice studies, no clinical data exist regarding the efficacy of manogepix, given alone or in combination for primary or salvage therapy of MCR. Recently, it was shown that *Rhizopus* hyphae killing can be enhanced by delivering sub-micromolar concentrations of amphotericin B through liposomes targeted to the fungal hyphae by the inclusion of dectin-1 receptor which binds to fungal β-glucans (Choudhury et al., 2022). These exciting results of enhancing the amphotericin B therapeutic index with lower and less toxic concentration are yet to be verified in animal models or clinical trials.

### Adjunctive therapies

Adjunctive therapies are crucial in managing mucormycosis. A promising adjunctive therapy is iron chelation, specifically with deferasirox, which has demonstrated potential in inhibiting the growth of Mucorales fungi by reducing the availability of iron, an essential nutrient for their proliferation (Ibrahim, 2006). Additionally, immune modulation therapies such as granulocyte transfusions and cytokine therapies are being studied to enhance the host’s immune response against Mucorales infections (Lanternier et al., 2015). While these adjunctive therapies show promise, further research and clinical trials are necessary to determine their optimal use and effectiveness. In individuals with DKA suspected of having MCR, the restoration of the host’s ability to chelate iron resulted in enhanced activity of neutrophils in killing Mucorales *ex vivo* (Gebremariam et al., 2016). This was achieved by reversing acidemia (acidosis) with sodium bicarbonate. Further, the administration of sodium bicarbonate partially prevented the capacity of *R. delemar* to invade endothelial cells (Gebremariam et al., 2016). Moreover, in a mouse model of ketoacidosis, treatment with sodium bicarbonate protected against invasive lung infection (Gebremariam et al., 2016). These findings suggest that individuals with DKA suspected of having Mucorales infection may benefit from the rapid correction of hyperglycemia and acidemia using insulin and sodium bicarbonate, respectively, to improve the host’s defense mechanisms (Gebremariam et al., 2016).

Restriction of available serum iron with new generation of xenosiderophores, inhibits fungal growth and protects DKA mice against MCR (Ibrahim, 2006; Ibrahim et al., 2007). In patients with DM it is suggested in case reports that it is beneficial to use iron chelation therapy as an adjunctive treatment (Spellberg, 2009). Adding deferasirox to L-AMB treatment was found to be harmful primarily in patients with hematologic malignancies in a small (20 patient) multi-center, placebo controlled, double-blind study (DEFEAT Mucor) (Spellberg et al., 2012a). The data do not support the use of deferasirox as an initial supplementary therapy for MCR in hematologic malignancies patients although the population imbalances in this small phase II study make generalizable inferences problematic. These findings are not surprising since hematologic malignancies patients generally do not suffer from iron overload due to acidosis or hyperglycemia.

The use of hyperbaric oxygen (HBO) is another therapy that is likely to be useful in conjunction with surgery and antifungal therapy. HBO therapy raises blood oxygen levels and boosts neutrophil activity (Lerche et al., 2022). Adjunctive HBO proved promising in diabetic patients (94% survival), but not in those with hematologic malignancies or bone marrow transplants (33% survival; *p* 0.02) (Price and Stevens, 1980; Ferguson et al., 1988; Kajs-Wyllie, 1995; Barratt et al., 2001; John et al., 2005). A better rate of survival was linked to prolonged courses of HBO (John et al., 2005).

Based on limited *in vitro* data and anecdotal case reports, strategies that boost the immune system, such as the administration of granulocyte (macrophage) colony stimulating factor or interferon-γ, or possibly check point inhibitors or their combination have been advocated as adjuvant therapy (Abzug and Walsh, 2004; Gil-Lamaignere et al., 2005). A combination of Interferon-γ with nivolumab (a monoclonal antibody that reduces programmed death-1 [PD-1] expression on T-cells) was found to be effective in an immunocompromised patient with intractable MCR in a recent case report (Grimaldi et al., 2017).

## MCR in the era of COVID-19

COVID-19-associated (CAM) MCR has recently emerged as an important superinfection among COVID-19 patients with documented cases from various regions of the world, and most notably in India (Raut and Huy, 2021; Ravani et al., 2021). Between May and August of 2021, more than 47,000 cases were reported, among mainly diabetic patients suffering from COVID-19 infection in India alone forcing the government to declare MCR as an epidemic. Whether COVID-19 infection by itself predisposes patients to MCR is not clear. Both in India and the rest of the world, the vast majority of excess cases of MCR during the COVID-19 pandemic have likely been attributable to a combination of DM and corticosteroid (John et al., 2021; Raut and Huy, 2021; Ravani et al., 2021; Singh et al., 2021). Almost 1/3 of the recent reported Indian MCR cases were among non-COVID-19 infected patients (Patel et al., 2021; Ravani et al., 2021), thereby underscoring the high baseline rate of infection in this country which was previously estimated to be 70-fold higher than any other part of the world (Patel et al., 2021). Furthermore, the large majority of MCR cases in COVID-19 patients in India have been of the rhino-orbital-cerebral (ROC) type (Ravani et al., 2021; Singh et al., 2021), and pulmonary infection has been rare. The pathogenesis of CAM remains an enigma. It is possible that COVID-19 predispose patients to newly onset DM or the ones with preexisting DM to experience worsening of glycemic control or full blown DKA, as SARS CoV-2 infection is associated with high expression of angiotensin-converting enzyme 2 (ACE2) receptor in pancreatic islets (potentially destroying these cells), along with increased insulin resistance due to cytokine storm (Kothandaraman et al., 2021). Interestingly, high expression of GRP78 in COVID-19 patients has been reported, possibly as a result of the viral-induced endoplasmic reticulum stress cascade (Sabirli et al., 2021). It was also recently shown that GRP78 forms a complex with ACE2 to act as an auxiliary receptor to the SARS COV-2 (Carlos et al., 2021). Thus, with GRP78 being a receptor to Mucorales fungi (Gebremariam et al., 2014; Gebremariam et al., 2016; Alqarihi et al., 2020), there is an increased probability that the presence of elevated GRP78 levels in COVID-19 patients specifically predispose to MCR.

## Summary

Despite advances in risk stratification, dissection of pathogenesis of the disease, imaging and increasingly the introduction of non-culture-based diagnostics, MCR continues to be associated with high rates of death and disability. Further improvements in molecular diagnostics and the establishment of large patient registries are key components of ongoing efforts. We believe that disease outcomes will further improve by the combination of much earlier diagnostics (surveillance *vs.* adjunct diagnostics), immuno-restoration/immunotherapeutic strategies, and the introduction of potent new antifungals. Further investments on developing pathophysiologically appropriate and phylogenetically disparate model systems of MCR (*e.g.* flies (Shirazi et al., 2014), Galleria mellonella (Maurer et al., 2019), zebra fish (Wurster et al., 2021) and mice (Jacobsen, 2019; Stevens et al., 2020)), along with advances in the molecular toolbox systems would further shed lights on the complex pathophysiology of this important disease.

## Author contributions

AA: Conceptualization, Writing – original draft, Writing – review & editing. DK: Writing – original draft, Writing – review & editing, Conceptualization, Funding acquisition, Supervision. AI: Conceptualization, Funding acquisition, Investigation, Supervision, Writing – original draft, Writing – review & editing
